# Supplementary material for: GRP78 regulates CD44v membrane homeostasis and cell spreading in tamoxifen-resistant breast cancer
Source: Life Sci Alliance. 2019 Aug 15;2(4):e201900377. doi: 10.26508/lsa.201900377 (PMC6696983; doi:10.26508/lsa.201900377)
Supplement: Supplementary file 1 [file LSA-2019-00377_Supplemental_Data_1.zip › CD44 sequencing results/Identification of CD44v-HA sequence.docx]

**>CD44v3-10_HA (Identified CD44 sequence with the COOH-terminal HA tag highlighted in yellow)**

atggacaagttttggtggcacgcagcctggggactctgcctcgtgccgctgagcctggcgcagatcgatttgaatataacctgccgctttgcaggtgtattccacgtggagaaaaatggtcgctacagcatctctcggacggaggccgctgacctctgcaaggctttcaatagcaccttgcccacaatggcccagatggagaaagctctgagcatcggatttgagacctgcaggtatgggttcatagaagggcacgtggtgattccccggatccaccccaactccatctgtgcagcaaacaacacaggggtgtacatcctcacatccaacacctcccagtatgacacatattgcttcaatgcttcagctccacctgaagaagattgtacatcagtcacagacctgcccaatgcctttgatggaccaattaccataactattgttaaccgtgatggcacccgctatgtccagaaaggagaatacagaacgaatcctgaagacatctaccccagcaaccctactgatgatgacgtgagcagcggctcctccagtgaaaggagcagcacttcaggaggttacatcttttacaccttttctactgtacaccccatcccagacgaagacagtccctggatcaccgacagcacagacagaatccctgctaccagtacgtcttcaaataccatctcagcaggctgggagccaaatgaagaaaatgaagatgaaagagacagacacctcagtttttctggatcaggcattgatgatgatgaagattttatctccagcaccatttcaaccacaccacgggcttttgaccacacaaaacagaaccaggactggacccagtggaacccaagccattcaaatccggaagtgctacttcagacaaccacaaggatgactgcagatgtagacagaaatggcaccactgcttatgaaggaaactggaacccagaagcacaccctcccctcattcaccatgagcatcatgaggaagaagagaccccacattctacaagcacaatccaggcaactcctagtagtacaacggaagaaacagctacccagaaggaacagtggtttggcaacagatggcatgagggatatcgccaaacacccaaagaagactcccattcgacaacagggacagctgcagcctcagctcataccagccatccaatgcaaggaaggacaacaccaagcccagaggacagttcctggactgatttcttcaacccaatctcacaccccatgggacgaggtcatcaagcaggaagaaggatggatatggactccagtcatagtataacgcttcagcctactgcaaatccaaacacaggtttggtggaagatttggacaggacaggacctctttcaatgacaacgcagcagagtaattctcagagcttctctacatcacatgaaggcttggaagaagataaagaccatccaacaacttctactctgacatcaagcaataggaatgatgtcacaggtggaagaagagacccaaatcattctgaaggctcaactactttactggaaggttatacctctcattacccacacacgaaggaaagcaggaccttcatcccagtgacctcagctaagactgggtcctttggagttactgcagttactgttggagattccaactctaatgtcaatcgttccttatcaggagaccaagacacattccaccccagtggggggtcccataccactcatggatctgaatcagatggacactcacatgggagtcaagaaggtggagcaaacacaacctctggtcctataaggacaccccaaattccagaatggctgatcatcttggcatccctcttggccttggctttgattcttgcagtttgcattgcagtcaacagtcgaagaaggtgtgggcagaagaaaaagctagtgatcaacagtggcaatggagctgtggaggacagaaagccaagtggactcaacggagaggccagcaagtctcaggaaatggtgcatttggtgaacaaggagtcgtcagaaactccagaccagtttatgacagctgatgagacaaggaacctgcagaatgtggacatgaagattggggtgTACCCATACGATGTTCCAGATTACGCTtaa

Note: Due to the length of CD44 coding sequence (CDS), we utilized 3 different primers to cover the entire length of CD44 CDS. The identified CD44 sequence contains variable exons 3 to 10. Below are the results of multiple sequence alignment between the HA-tagged full-length CD44v3-10 and the sequencing results. Text highlighted in green indicates the start or end site of reliable sequencing trace.

**>vHA_CMV-Forward_F06.ab1**

NNNNNNNNNNNNNNNNGNAGAGCTCTCTGGCTAACTAGAGAACCCACTGCTTACTGGCTTATCGAAATTAATACGACTCACTATAGGGAGACCCAAGCTTGGTACCATGGACAAGTTTTGGTGGCACGCAGCCTGGGGACTCTGCCTCGTGCCGCTGAGCCTGGCGCAGATCGATTTGAATATAACCTGCCGCTTTGCAGGTGTATTCCACGTGGAGAAAAATGGTCGCTACAGCATCTCTCGGACGGAGGCCGCTGACCTCTGCAAGGCTTTCAATAGCACCTTGCCCACAATGGCCCAGATGGAGAAAGCTCTGAGCATCGGATTTGAGACCTGCAGGTATGGGTTCATAGAAGGGCACGTGGTGATTCCCCGGATCCACCCCAACTCCATCTGTGCAGCAAACAACACAGGGGTGTACATCCTCACATCCAACACCTCCCAGTATGACACATATTGCTTCAATGCTTCAGCTCCACCTGAAGAAGATTGTACATCAGTCACAGACCTGCCCAATGCCTTTGATGGACCAATTACCATAACTATTGTTAACCGTGATGGCACCCGCTATGTCCAGAAAGGAGAATACAGAACGAATCCTGAAGACATCTACCCCAGCAACCCTACTGATGATGACGTGAGCAGCGGCTCCTCCAGTGAAAGGAGCAGCACTTCAGGAGGTTACATCTTTTACACCTTTTCTACTGTACACCCCATCCCAGACGAAGACAGTCCCTGGATCACCGACAGCACAGACAGAATCCCTGCTACCAGTACGTCTTCAAATACCATCTCAGCAGGCTGGGAGCCAAATGAAGAAAATGAAGATGAAAGAGACAGACACCTCAGTTTTTCTGGATCNGGCATTGATGATGATGAAGATTTTATCTCCAGCACCATTTCAACCACACCACGGGCTTTTGACCACACAAAACAGAACCAGGACTGGACCCAGTGGNACCCAAGCCATTCAAATCCCGGAAGTGCTACTTCAGACACCACANNNTGACTGCAGANGNNNACNGAAATGGCACNCTGCTNNNNNNNAANTGGAACCCANANCNNNNCCCTCCCCNCATCNNCNTGANCATCATGNNNGANANANACCCCNNNTTCTNNNAGCNN

CD44v3-10_HA ------------------------------------------------------------ 0

vHA_CMV-Forward_F06.ab1 NNNNNNNNNNNNNNNNGNAGAGCTCTCTGGCTAACTAGAGAACCCACTGCTTACTGGCTT 60

CD44v3-10_HA ----------------------------------------------atggacaagttttg 14

vHA_CMV-Forward_F06.ab1 ATCGAAATTAATACGACTCACTATAGGGAGACCCAAGCTTGGTACCATGGACAAGTTTTG 120

**************

CD44v3-10_HA gtggcacgcagcctggggactctgcctcgtgccgctgagcctggcgcagatcgatttgaa 74

vHA_CMV-Forward_F06.ab1 GTGGCACGCAGCCTGGGGACTCTGCCTCGTGCCGCTGAGCCTGGCGCAGATCGATTTGAA 180

************************************************************

CD44v3-10_HA tataacctgccgctttgcaggtgtattccacgtggagaaaaatggtcgctacagcatctc 134

vHA_CMV-Forward_F06.ab1 TATAACCTGCCGCTTTGCAGGTGTATTCCACGTGGAGAAAAATGGTCGCTACAGCATCTC 240

************************************************************

CD44v3-10_HA tcggacggaggccgctgacctctgcaaggctttcaatagcaccttgcccacaatggccca 194

vHA_CMV-Forward_F06.ab1 TCGGACGGAGGCCGCTGACCTCTGCAAGGCTTTCAATAGCACCTTGCCCACAATGGCCCA 300

************************************************************

CD44v3-10_HA gatggagaaagctctgagcatcggatttgagacctgcaggtatgggttcatagaagggca 254

vHA_CMV-Forward_F06.ab1 GATGGAGAAAGCTCTGAGCATCGGATTTGAGACCTGCAGGTATGGGTTCATAGAAGGGCA 360

************************************************************

CD44v3-10_HA cgtggtgattccccggatccaccccaactccatctgtgcagcaaacaacacaggggtgta 314

vHA_CMV-Forward_F06.ab1 CGTGGTGATTCCCCGGATCCACCCCAACTCCATCTGTGCAGCAAACAACACAGGGGTGTA 420

************************************************************

CD44v3-10_HA catcctcacatccaacacctcccagtatgacacatattgcttcaatgcttcagctccacc 374

vHA_CMV-Forward_F06.ab1 CATCCTCACATCCAACACCTCCCAGTATGACACATATTGCTTCAATGCTTCAGCTCCACC 480

************************************************************

CD44v3-10_HA tgaagaagattgtacatcagtcacagacctgcccaatgcctttgatggaccaattaccat 434

vHA_CMV-Forward_F06.ab1 TGAAGAAGATTGTACATCAGTCACAGACCTGCCCAATGCCTTTGATGGACCAATTACCAT 540

************************************************************

CD44v3-10_HA aactattgttaaccgtgatggcacccgctatgtccagaaaggagaatacagaacgaatcc 494

vHA_CMV-Forward_F06.ab1 AACTATTGTTAACCGTGATGGCACCCGCTATGTCCAGAAAGGAGAATACAGAACGAATCC 600

************************************************************

CD44v3-10_HA tgaagacatctaccccagcaaccctactgatgatgacgtgagcagcggctcctccagtga 554

vHA_CMV-Forward_F06.ab1 TGAAGACATCTACCCCAGCAACCCTACTGATGATGACGTGAGCAGCGGCTCCTCCAGTGA 660

************************************************************

CD44v3-10_HA aaggagcagcacttcaggaggttacatcttttacaccttttctactgtacaccccatccc 614

vHA_CMV-Forward_F06.ab1 AAGGAGCAGCACTTCAGGAGGTTACATCTTTTACACCTTTTCTACTGTACACCCCATCCC 720

************************************************************

CD44v3-10_HA agacgaagacagtccctggatcaccgacagcacagacagaatccctgctaccagtacgtc 674

vHA_CMV-Forward_F06.ab1 AGACGAAGACAGTCCCTGGATCACCGACAGCACAGACAGAATCCCTGCTACCAGTACGTC 780

************************************************************

CD44v3-10_HA ttcaaataccatctcagcaggctgggagccaaatgaagaaaatgaagatgaaagagacag 734

vHA_CMV-Forward_F06.ab1 TTCAAATACCATCTCAGCAGGCTGGGAGCCAAATGAAGAAAATGAAGATGAAAGAGACAG 840

************************************************************

CD44v3-10_HA acacctcagtttttctggatcaggcattgatgatgatgaagattttatctccagcaccat 794

vHA_CMV-Forward_F06.ab1 ACACCTCAGTTTTTCTGGATCNGGCATTGATGATGATGAAGATTTTATCTCCAGCACCAT 900

********************* **************************************

CD44v3-10_HA ttcaaccacaccacgggcttttgaccacacaaaacagaaccaggactggacccagtggaa 854

vHA_CMV-Forward_F06.ab1 TTCAACCACACCACGGGCTTTTGACCACACAAAACAGAACCAGGACTGGACCCAGTGGNA 960

********************************************************** *

CD44v3-10_HA cccaagccattcaaatccggaagtgcta-cttcagacaaccacaaggatgactgcagatg 913

vHA_CMV-Forward_F06.ab1 CCCAAGCCATTCAAATCCCGGAAGTGCTACTTCAGACACCAC--ANNNTGACTGCAGANG 1018

****************** * * ********* * * ********** *

CD44v3-10_HA tagacagaaatggcaccactgcttatgaaggaaactggaacccagaagcacaccctcccc 973

vHA_CMV-Forward_F06.ab1 NNNACNGAAATGGCACNCTGCTNN---NNNNNAANTGGAACCCANANCNNNNCCCTCCCC 1075

** ********** ** ********* * ********

CD44v3-10_HA tcattcaccatgagcatcatgaggaagaagagaccccacattctacaagcacaatccagg 1033

vHA_CMV-Forward_F06.ab1 N-CATCNNCNTGANCATCATGNNNGANANANACC-CCNNNTTCTNNNAGCNN-------- 1125

** * *** ******* * * * ** **** ***

CD44v3-10_HA caactcctagtagtacaacggaagaaacagctacccagaaggaacagtggtttggcaaca 1093

vHA_CMV-Forward_F06.ab1 ------------------------------------------------------------ 1125

CD44v3-10_HA gatggcatgagggatatcgccaaacacccaaagaagactcccattcgacaacagggacag 1153

vHA_CMV-Forward_F06.ab1 ------------------------------------------------------------ 1125

CD44v3-10_HA ctgcagcctcagctcataccagccatccaatgcaaggaaggacaacaccaagcccagagg 1213

vHA_CMV-Forward_F06.ab1 ------------------------------------------------------------ 1125

CD44v3-10_HA acagttcctggactgatttcttcaacccaatctcacaccccatgggacgaggtcatcaag 1273

vHA_CMV-Forward_F06.ab1 ------------------------------------------------------------ 1125

CD44v3-10_HA caggaagaaggatggatatggactccagtcatagtataacgcttcagcctactgcaaatc 1333

vHA_CMV-Forward_F06.ab1 ------------------------------------------------------------ 1125

CD44v3-10_HA caaacacaggtttggtggaagatttggacaggacaggacctctttcaatgacaacgcagc 1393

vHA_CMV-Forward_F06.ab1 ------------------------------------------------------------ 1125

CD44v3-10_HA agagtaattctcagagcttctctacatcacatgaaggcttggaagaagataaagaccatc 1453

vHA_CMV-Forward_F06.ab1 ------------------------------------------------------------ 1125

CD44v3-10_HA caacaacttctactctgacatcaagcaataggaatgatgtcacaggtggaagaagagacc 1513

vHA_CMV-Forward_F06.ab1 ------------------------------------------------------------ 1125

CD44v3-10_HA caaatcattctgaaggctcaactactttactggaaggttatacctctcattacccacaca 1573

vHA_CMV-Forward_F06.ab1 ------------------------------------------------------------ 1125

CD44v3-10_HA cgaaggaaagcaggaccttcatcccagtgacctcagctaagactgggtcctttggagtta 1633

vHA_CMV-Forward_F06.ab1 ------------------------------------------------------------ 1125

CD44v3-10_HA ctgcagttactgttggagattccaactctaatgtcaatcgttccttatcaggagaccaag 1693

vHA_CMV-Forward_F06.ab1 ------------------------------------------------------------ 1125

CD44v3-10_HA acacattccaccccagtggggggtcccataccactcatggatctgaatcagatggacact 1753

vHA_CMV-Forward_F06.ab1 ------------------------------------------------------------ 1125

CD44v3-10_HA cacatgggagtcaagaaggtggagcaaacacaacctctggtcctataaggacaccccaaa 1813

vHA_CMV-Forward_F06.ab1 ------------------------------------------------------------ 1125

CD44v3-10_HA ttccagaatggctgatcatcttggcatccctcttggccttggctttgattcttgcagttt 1873

vHA_CMV-Forward_F06.ab1 ------------------------------------------------------------ 1125

CD44v3-10_HA gcattgcagtcaacagtcgaagaaggtgtgggcagaagaaaaagctagtgatcaacagtg 1933

vHA_CMV-Forward_F06.ab1 ------------------------------------------------------------ 1125

CD44v3-10_HA gcaatggagctgtggaggacagaaagccaagtggactcaacggagaggccagcaagtctc 1993

vHA_CMV-Forward_F06.ab1 ------------------------------------------------------------ 1125

CD44v3-10_HA aggaaatggtgcatttggtgaacaaggagtcgtcagaaactccagaccagtttatgacag 2053

vHA_CMV-Forward_F06.ab1 ------------------------------------------------------------ 1125

CD44v3-10_HA ctgatgagacaaggaacctgcagaatgtggacatgaagattggggtgTACCCATACGATG 2113

vHA_CMV-Forward_F06.ab1 ------------------------------------------------------------ 1125

CD44v3-10_HA TTCCAGATTACGCTtaa 2130

vHA_CMV-Forward_F06.ab1 ----------------- 1125

**>vHA-sF_C05.ab1**

NNNNNNNNNNNNNNNNNNNCTTTTACNCCTTTTCTACTGTACACCCCATCCCAGACGAAGACAGTCCCTGGATCACCGACAGCACAGACAGAATCCCTGCTACCAGTACGTCTTCAAATACCATCTCAGCAGGCTGGGAGCCAAATGAAGAAAATGAAGATGAAAGAGACAGACACCTCAGTTTTTCTGGATCAGGCATTGATGATGATGAAGATTTTATCTCCAGCACCATTTCAACCACACCACGGGCTTTTGACCACACAAAACAGAACCAGGACTGGACCCAGTGGAACCCAAGCCATTCAAATCCGGAAGTGCTACTTCAGACAACCACAAGGATGACTGCAGATGTAGACAGAAATGGCACCACTGCTTATGAAGGAAACTGGAACCCAGAAGCACACCCTCCCCTCATTCACCATGAGCATCATGAGGAAGAAGAGACCCCACATTCTACAAGCACAATCCAGGCAACTCCTAGTAGTACAACGGAAGAAACAGCTACCCAGAAGGAACAGTGGTTTGGCAACAGATGGCATGAGGGATATCGCCAAACACCCAAAGAAGACTCCCATTCGACAACAGGGACAGCTGCAGCCTCAGCTCATACCAGCCATCCAATGCAAGGAAGGACAACACCAAGCCCAGAGGACAGTTCCTGGACTGATTTCTTCAACCCAATCTCACACCCCATGGGACGAGGTCATCAAGCAGGAAGAAGGATGGATATGGACTCCAGTCATAGTATAACGCTTCAGCCTACTGCAAATCCAAACACAGGTTTGGTGGAAGATTTGGACAGGACAGGACCTCTTTCAATGACAACGCAGCAGAGTAATTCTCAGAGCTTCTCTACATCACATGAANGGCTTGGGAAGAAGATAAAGACCATCCAACAACTTCTACTCTGACATCAAGCAATANGAATGATGTCACAGGTGGAAGAAGAGACCCAAATCATTCTGAAGGNTCAACTACTTTACTGGNAGGTTATACCTCTCATTACCCACACACGAAGGAAAGCAGGANCTTCATCCCAGTGACNCAGCTANACTGGGTCCTTTNGANTTACTGCANNNCTNNNNAGATTCNNCTCTAANGTCAATCNNNNNNCNGNNANNNANNCNTTCNACCCCCNNNNGGGGGNCCNTNCNNNCANNNNNTNANNNNNGNNNNNNNNNNGNNNNNNNNNNNNNGNANNNANNNNNNNNNNNNNNNNNNNNNNNNCCCNATTCN

CD44v3-10_HA atggacaagttttggtggcacgcagcctggggactctgcctcgtgccgctgagcctggcg 60

vHA-sF_C05.ab1 ------------------------------------------------------------ 0

CD44v3-10_HA cagatcgatttgaatataacctgccgctttgcaggtgtattccacgtggagaaaaatggt 120

vHA-sF_C05.ab1 ------------------------------------------------------------ 0

CD44v3-10_HA cgctacagcatctctcggacggaggccgctgacctctgcaaggctttcaatagcaccttg 180

vHA-sF_C05.ab1 ------------------------------------------------------------ 0

CD44v3-10_HA cccacaatggcccagatggagaaagctctgagcatcggatttgagacctgcaggtatggg 240

vHA-sF_C05.ab1 ------------------------------------------------------------ 0

CD44v3-10_HA ttcatagaagggcacgtggtgattccccggatccaccccaactccatctgtgcagcaaac 300

vHA-sF_C05.ab1 ------------------------------------------------------------ 0

CD44v3-10_HA aacacaggggtgtacatcctcacatccaacacctcccagtatgacacatattgcttcaat 360

vHA-sF_C05.ab1 ------------------------------------------------------------ 0

CD44v3-10_HA gcttcagctccacctgaagaagattgtacatcagtcacagacctgcccaatgcctttgat 420

vHA-sF_C05.ab1 ------------------------------------------------------------ 0

CD44v3-10_HA ggaccaattaccataactattgttaaccgtgatggcacccgctatgtccagaaaggagaa 480

vHA-sF_C05.ab1 ------------------------------------------------------------ 0

CD44v3-10_HA tacagaacgaatcctgaagacatctaccccagcaaccctactgatgatgacgtgagcagc 540

vHA-sF_C05.ab1 ------------------------------------------------------------ 0

CD44v3-10_HA ggctcctccagtgaaaggagcagcacttcaggaggttacatcttttacaccttttctact 600

vHA-sF_C05.ab1 ----------------------NNNNNNNNNNNNNNNNNNNCTTTTACNCCTTTTCTACT 38

******* ***********

CD44v3-10_HA gtacaccccatcccagacgaagacagtccctggatcaccgacagcacagacagaatccct 660

vHA-sF_C05.ab1 GTACACCCCATCCCAGACGAAGACAGTCCCTGGATCACCGACAGCACAGACAGAATCCCT 98

************************************************************

CD44v3-10_HA gctaccagtacgtcttcaaataccatctcagcaggctgggagccaaatgaagaaaatgaa 720

vHA-sF_C05.ab1 GCTACCAGTACGTCTTCAAATACCATCTCAGCAGGCTGGGAGCCAAATGAAGAAAATGAA 158

************************************************************

CD44v3-10_HA gatgaaagagacagacacctcagtttttctggatcaggcattgatgatgatgaagatttt 780

vHA-sF_C05.ab1 GATGAAAGAGACAGACACCTCAGTTTTTCTGGATCAGGCATTGATGATGATGAAGATTTT 218

************************************************************

CD44v3-10_HA atctccagcaccatttcaaccacaccacgggcttttgaccacacaaaacagaaccaggac 840

vHA-sF_C05.ab1 ATCTCCAGCACCATTTCAACCACACCACGGGCTTTTGACCACACAAAACAGAACCAGGAC 278

************************************************************

CD44v3-10_HA tggacccagtggaacccaagccattcaaatccggaagtgctacttcagacaaccacaagg 900

vHA-sF_C05.ab1 TGGACCCAGTGGAACCCAAGCCATTCAAATCCGGAAGTGCTACTTCAGACAACCACAAGG 338

************************************************************

CD44v3-10_HA atgactgcagatgtagacagaaatggcaccactgcttatgaaggaaactggaacccagaa 960

vHA-sF_C05.ab1 ATGACTGCAGATGTAGACAGAAATGGCACCACTGCTTATGAAGGAAACTGGAACCCAGAA 398

************************************************************

CD44v3-10_HA gcacaccctcccctcattcaccatgagcatcatgaggaagaagagaccccacattctaca 1020

vHA-sF_C05.ab1 GCACACCCTCCCCTCATTCACCATGAGCATCATGAGGAAGAAGAGACCCCACATTCTACA 458

************************************************************

CD44v3-10_HA agcacaatccaggcaactcctagtagtacaacggaagaaacagctacccagaaggaacag 1080

vHA-sF_C05.ab1 AGCACAATCCAGGCAACTCCTAGTAGTACAACGGAAGAAACAGCTACCCAGAAGGAACAG 518

************************************************************

CD44v3-10_HA tggtttggcaacagatggcatgagggatatcgccaaacacccaaagaagactcccattcg 1140

vHA-sF_C05.ab1 TGGTTTGGCAACAGATGGCATGAGGGATATCGCCAAACACCCAAAGAAGACTCCCATTCG 578

************************************************************

CD44v3-10_HA acaacagggacagctgcagcctcagctcataccagccatccaatgcaaggaaggacaaca 1200

vHA-sF_C05.ab1 ACAACAGGGACAGCTGCAGCCTCAGCTCATACCAGCCATCCAATGCAAGGAAGGACAACA 638

************************************************************

CD44v3-10_HA ccaagcccagaggacagttcctggactgatttcttcaacccaatctcacaccccatggga 1260

vHA-sF_C05.ab1 CCAAGCCCAGAGGACAGTTCCTGGACTGATTTCTTCAACCCAATCTCACACCCCATGGGA 698

************************************************************

CD44v3-10_HA cgaggtcatcaagcaggaagaaggatggatatggactccagtcatagtataacgcttcag 1320

vHA-sF_C05.ab1 CGAGGTCATCAAGCAGGAAGAAGGATGGATATGGACTCCAGTCATAGTATAACGCTTCAG 758

************************************************************

CD44v3-10_HA cctactgcaaatccaaacacaggtttggtggaagatttggacaggacaggacctctttca 1380

vHA-sF_C05.ab1 CCTACTGCAAATCCAAACACAGGTTTGGTGGAAGATTTGGACAGGACAGGACCTCTTTCA 818

************************************************************

CD44v3-10_HA atgacaacgcagcagagtaattctcagagcttctctacatcacatgaaggct--tggaag 1438

vHA-sF_C05.ab1 ATGACAACGCAGCAGAGTAATTCTCAGAGCTTCTCTACATCACATGAANGGCTTGGGAAG 878

************************************************ * *****

CD44v3-10_HA aagataaagaccatccaacaacttctactctgacatcaagcaataggaatgatgtcacag 1498

vHA-sF_C05.ab1 AAGATAAAGACCATCCAACAACTTCTACTCTGACATCAAGCAATANGAATGATGTCACAG 938

********************************************* **************

CD44v3-10_HA gtggaagaagagacccaaatcattctgaaggctcaactactttactggaaggttatacct 1558

vHA-sF_C05.ab1 GTGGAAGAAGAGACCCAAATCATTCTGAAGGNTCAACTACTTTACTGGNAGGTTATACCT 998

******************************* **************** ***********

CD44v3-10_HA ctcattacccacacacgaaggaaagcaggaccttcatcccagtgacctcagctaagactg 1618

vHA-sF_C05.ab1 CTCATTACCCACACACGAAGGAAAGCAGGANCTTCATCCCAGTGACNCAGCTANA--CTG 1056

****************************** *************** * ***

CD44v3-10_HA ggtcctttggagttactgcagttactgttggagattccaactctaatgtcaatcgttcct 1678

vHA-sF_C05.ab1 GGTCCTTTNGANTTACTGCANNNCTNNNNAGA---TTCNNCTCTAANGTCAATCNNNNNN 1113

******** ** ******** ** * * ****** *******

CD44v3-10_HA tatcaggagaccaagacacattccaccccagtggggggtcccataccactcatggatctg 1738

vHA-sF_C05.ab1 CNGNN------ANNNANNCNTTCNACCCCCNNNNGGGGGNCCNTNCNNNCANNNNNTNAN 1167

* * *** ***** **** ** * * *

CD44v3-10_HA aatcagatggacactcacatgggagtcaagaaggtggagcaaacacaacctctggtccta 1798

vHA-sF_C05.ab1 NNNNGNNNNNNNNNNGNNNNNNNN------NNNNNGNANN---NANNNNNNNNNNNNNNN 1218

* * *

CD44v3-10_HA taaggacaccccaaattccagaatggctgatcatcttggcatccctcttggccttggctt 1858

vHA-sF_C05.ab1 N-NNNNNNNNCCCNATTCN----------------------------------------- 1236

** ****

CD44v3-10_HA tgattcttgcagtttgcattgcagtcaacagtcgaagaaggtgtgggcagaagaaaaagc 1918

vHA-sF_C05.ab1 ------------------------------------------------------------ 1236

CD44v3-10_HA tagtgatcaacagtggcaatggagctgtggaggacagaaagccaagtggactcaacggag 1978

vHA-sF_C05.ab1 ------------------------------------------------------------ 1236

CD44v3-10_HA aggccagcaagtctcaggaaatggtgcatttggtgaacaaggagtcgtcagaaactccag 2038

vHA-sF_C05.ab1 ------------------------------------------------------------ 1236

CD44v3-10_HA accagtttatgacagctgatgagacaaggaacctgcagaatgtggacatgaagattgggg 2098

vHA-sF_C05.ab1 ------------------------------------------------------------ 1236

CD44v3-10_HA tgTACCCATACGATGTTCCAGATTACGCTtaa 2130

vHA-sF_C05.ab1 -------------------------------- 1236

**>vHA-V4_C02.ab1**

NNNNNNNNNNNNNNNGGNACCACTGCTTATGAGGANNTGGAACCCAGAAGCACACCCTCCCCTCATTCACCATGAGCATCATGAGGAAGAAGAGACCCCACATTCTACAAGCACAATCCAGGCAACTCCTAGTAGTACAACGGAAGAAACAGCTACCCAGAAGGAACAGTGGTTTGGCAACAGATGGCATGAGGGATATCGCCAAACACCCAAAGAAGACTCCCATTCGACAACAGGGACAGCTGCAGCCTCAGCTCATACCAGCCATCCAATGCAAGGAAGGACAACACCAAGCCCAGAGGACAGTTCCTGGACTGATTTCTTCAACCCAATCTCACACCCCATGGGACGAGGTCATCAAGCAGGAAGAAGGATGGATATGGACTCCAGTCATAGTATAACGCTTCAGCCTACTGCAAATCCAAACACAGGTTTGGTGGAAGATTTGGACAGGACAGGACCTCTTTCAATGACAACGCAGCAGAGTAATTCTCAGAGCTTCTCTACATCACATGAAGGCTTGGAAGAAGATAAAGACCATCCAACAACTTCTACTCTGACATCAAGCAATAGGAATGATGTCACAGGTGGAAGAAGAGACCCAAATCATTCTGAAGGCTCAACTACTTTACTGGAAGGTTATACCTCTCATTACCCACACACGAAGGAAAGCAGGACCTTCATCCCAGTGACCTCAGCTAAGACTGGGTCCTTTGGAGTTACTGCAGTTACTGTTGGAGATTCCAACTCTAATGTCAATCGTTCCTTATCAGGAGACCAAGACACATTCCACCCCAGTGGGGGGGTCCCATACCACTCATGGATCTGAATCAGATGGACACTCACATGGGAGTCAAGAANNNGGAGCAAACACAACCTCTGGTCCTATAANGACACCCCAAATTCCAGAATGGCTGATCATCTTGGCATCCCTCTTGGCCTTGGCTTTGATTCTTGCAGTTTGCATTGCAGTCAACAGTCGAAGAANNGTGGGCAGAANAAAAGCTAGTGATCANNGTGGCAATGNNGCTGNNGNNNNAGAAAGCCAGNNNNTNANGNNNGNCAGCAGTCTNNNNNTGGNGCATTTNNNGANNNNNNNNTCNN

CD44v3-10_HA atggacaagttttggtggcacgcagcctggggactctgcctcgtgccgctgagcctggcg 60

vHA-V4_C02.ab1 ------------------------------------------------------------ 0

CD44v3-10_HA cagatcgatttgaatataacctgccgctttgcaggtgtattccacgtggagaaaaatggt 120

vHA-V4_C02.ab1 ------------------------------------------------------------ 0

CD44v3-10_HA cgctacagcatctctcggacggaggccgctgacctctgcaaggctttcaatagcaccttg 180

vHA-V4_C02.ab1 ------------------------------------------------------------ 0

CD44v3-10_HA cccacaatggcccagatggagaaagctctgagcatcggatttgagacctgcaggtatggg 240

vHA-V4_C02.ab1 ------------------------------------------------------------ 0

CD44v3-10_HA ttcatagaagggcacgtggtgattccccggatccaccccaactccatctgtgcagcaaac 300

vHA-V4_C02.ab1 ------------------------------------------------------------ 0

CD44v3-10_HA aacacaggggtgtacatcctcacatccaacacctcccagtatgacacatattgcttcaat 360

vHA-V4_C02.ab1 ------------------------------------------------------------ 0

CD44v3-10_HA gcttcagctccacctgaagaagattgtacatcagtcacagacctgcccaatgcctttgat 420

vHA-V4_C02.ab1 ------------------------------------------------------------ 0

CD44v3-10_HA ggaccaattaccataactattgttaaccgtgatggcacccgctatgtccagaaaggagaa 480

vHA-V4_C02.ab1 ------------------------------------------------------------ 0

CD44v3-10_HA tacagaacgaatcctgaagacatctaccccagcaaccctactgatgatgacgtgagcagc 540

vHA-V4_C02.ab1 ------------------------------------------------------------ 0

CD44v3-10_HA ggctcctccagtgaaaggagcagcacttcaggaggttacatcttttacaccttttctact 600

vHA-V4_C02.ab1 ------------------------------------------------------------ 0

CD44v3-10_HA gtacaccccatcccagacgaagacagtccctggatcaccgacagcacagacagaatccct 660

vHA-V4_C02.ab1 ------------------------------------------------------------ 0

CD44v3-10_HA gctaccagtacgtcttcaaataccatctcagcaggctgggagccaaatgaagaaaatgaa 720

vHA-V4_C02.ab1 ------------------------------------------------------------ 0

CD44v3-10_HA gatgaaagagacagacacctcagtttttctggatcaggcattgatgatgatgaagatttt 780

vHA-V4_C02.ab1 ------------------------------------------------------------ 0

CD44v3-10_HA atctccagcaccatttcaaccacaccacgggcttttgaccacacaaaacagaaccaggac 840

vHA-V4_C02.ab1 ------------------------------------------------------------ 0

CD44v3-10_HA tggacccagtggaacccaagccattcaaatccggaagtgctacttcagacaaccacaagg 900

vHA-V4_C02.ab1 ------------------------------------------------------------ 0

CD44v3-10_HA atgactgcagatgtagacagaaatggcaccactgcttatgaaggaaactggaacccagaa 960

vHA-V4_C02.ab1 ---------NNNNNNNNNNNNNNNGGNACCAC--TGCTTATGAGGANNTGGAACCCAGAA 49

** ***** * * * ************

CD44v3-10_HA gcacaccctcccctcattcaccatgagcatcatgaggaagaagagaccccacattctaca 1020

vHA-V4_C02.ab1 GCACACCCTCCCCTCATTCACCATGAGCATCATGAGGAAGAAGAGACCCCACATTCTACA 109

************************************************************

CD44v3-10_HA agcacaatccaggcaactcctagtagtacaacggaagaaacagctacccagaaggaacag 1080

vHA-V4_C02.ab1 AGCACAATCCAGGCAACTCCTAGTAGTACAACGGAAGAAACAGCTACCCAGAAGGAACAG 169

************************************************************

CD44v3-10_HA tggtttggcaacagatggcatgagggatatcgccaaacacccaaagaagactcccattcg 1140

vHA-V4_C02.ab1 TGGTTTGGCAACAGATGGCATGAGGGATATCGCCAAACACCCAAAGAAGACTCCCATTCG 229

************************************************************

CD44v3-10_HA acaacagggacagctgcagcctcagctcataccagccatccaatgcaaggaaggacaaca 1200

vHA-V4_C02.ab1 ACAACAGGGACAGCTGCAGCCTCAGCTCATACCAGCCATCCAATGCAAGGAAGGACAACA 289

************************************************************

CD44v3-10_HA ccaagcccagaggacagttcctggactgatttcttcaacccaatctcacaccccatggga 1260

vHA-V4_C02.ab1 CCAAGCCCAGAGGACAGTTCCTGGACTGATTTCTTCAACCCAATCTCACACCCCATGGGA 349

************************************************************

CD44v3-10_HA cgaggtcatcaagcaggaagaaggatggatatggactccagtcatagtataacgcttcag 1320

vHA-V4_C02.ab1 CGAGGTCATCAAGCAGGAAGAAGGATGGATATGGACTCCAGTCATAGTATAACGCTTCAG 409

************************************************************

CD44v3-10_HA cctactgcaaatccaaacacaggtttggtggaagatttggacaggacaggacctctttca 1380

vHA-V4_C02.ab1 CCTACTGCAAATCCAAACACAGGTTTGGTGGAAGATTTGGACAGGACAGGACCTCTTTCA 469

************************************************************

CD44v3-10_HA atgacaacgcagcagagtaattctcagagcttctctacatcacatgaaggcttggaagaa 1440

vHA-V4_C02.ab1 ATGACAACGCAGCAGAGTAATTCTCAGAGCTTCTCTACATCACATGAAGGCTTGGAAGAA 529

************************************************************

CD44v3-10_HA gataaagaccatccaacaacttctactctgacatcaagcaataggaatgatgtcacaggt 1500

vHA-V4_C02.ab1 GATAAAGACCATCCAACAACTTCTACTCTGACATCAAGCAATAGGAATGATGTCACAGGT 589

************************************************************

CD44v3-10_HA ggaagaagagacccaaatcattctgaaggctcaactactttactggaaggttatacctct 1560

vHA-V4_C02.ab1 GGAAGAAGAGACCCAAATCATTCTGAAGGCTCAACTACTTTACTGGAAGGTTATACCTCT 649

************************************************************

CD44v3-10_HA cattacccacacacgaaggaaagcaggaccttcatcccagtgacctcagctaagactggg 1620

vHA-V4_C02.ab1 CATTACCCACACACGAAGGAAAGCAGGACCTTCATCCCAGTGACCTCAGCTAAGACTGGG 709

************************************************************

CD44v3-10_HA tcctttggagttactgcagttactgttggagattccaactctaatgtcaatcgttcctta 1680

vHA-V4_C02.ab1 TCCTTTGGAGTTACTGCAGTTACTGTTGGAGATTCCAACTCTAATGTCAATCGTTCCTTA 769

************************************************************

CD44v3-10_HA tcaggagaccaagacacattccaccccagtgg-ggggtcccataccactcatggatctga 1739

vHA-V4_C02.ab1 TCAGGAGACCAAGACACATTCCACCCCAGTGGGGGGGTCCCATACCACTCATGGATCTGA 829

******************************** ***************************

CD44v3-10_HA atcagatggacactcacatgggagtcaagaaggtggagcaaacacaacctctggtcctat 1799

vHA-V4_C02.ab1 ATCAGATGGACACTCACATGGGAGTCAAGAANNNGGAGCAAACACAACCTCTGGTCCTAT 889

******************************* **************************

CD44v3-10_HA aaggacaccccaaattccagaatggctgatcatcttggcatccctcttggccttggcttt 1859

vHA-V4_C02.ab1 AANGACACCCCAAATTCCAGAATGGCTGATCATCTTGGCATCCCTCTTGGCCTTGGCTTT 949

** *********************************************************

CD44v3-10_HA gattcttgcagtttgcattgcagtcaacagtcgaagaaggtgtgggcagaagaaaaagct 1919

vHA-V4_C02.ab1 GATTCTTGCAGTTTGCATTGCAGTCAACAGTCGAAGAANNGTGGGCAGAANAAAAGC--T 1007

************************************** ** * *** *

CD44v3-10_HA agtgatcaacagtggcaatggagctgtggaggacagaaagccaagtggactcaacggaga 1979

vHA-V4_C02.ab1 AGTGATCANN-GTGGCAATGNNGCTGNNGNNNNAGAAAGC-CAGNNNNTNAN-------- 1057

******** ********* **** * ** **

CD44v3-10_HA ggccagcaagtctcaggaaatggtgcatttggtgaacaaggagtcgtcagaaactccaga 2039

vHA-V4_C02.ab1 GNNNGNCAGCAGTCTNNNNNTGGNGCATTTNNNGANNNNNNNNTCNN------------- 1104

* ** ** *** ****** ** **

CD44v3-10_HA ccagtttatgacagctgatgagacaaggaacctgcagaatgtggacatgaagattggggt 2099

vHA-V4_C02.ab1 ------------------------------------------------------------ 1104

CD44v3-10_HA gTACCCATACGATGTTCCAGATTACGCTtaa 2130

vHA-V4_C02.ab1 ------------------------------- 1104

**>vHA-SP6_R_F05.ab1**

NNNNNNNNNNNTGNNTGNTCGAGCGGCGCCAGTGTGATGGATATCTGCAGAATTCTTAAGCGTAATCTGGAACATCGTATGGGTACACCCCAATCTTCATGTCCACATTCTGCAGGTTCCTTGTCTCATCAGCTGTCATAAACTGGTCTGGAGTTTCTGACGACTCCTTGTTCACCAAATGCACCATTTCCTGAGACTTGCTGGCCTCTCCGTTGAGTCCACTTGGCTTTCTGTCCTCCACAGCTCCATTGCCACTGTTGATCACTAGCTTTTTCTTCTGCCCACACCTTCTTCGACTGTTGACTGCAATGCAAACTGCAAGAATCAAAGCCAAGGCCAAGAGGGATGCCAAGATGATCAGCCATTCTGGAATTTGGGGTGTCCTTATAGGACCAGAGGTTGTGTTTGCTCCACCTTCTTGACTCCCATGTGAGTGTCCATCTGATTCAGATCCATGAGTGGTATGGGACCCCCCACTGGGGTGGAATGTGTCTTGGTCTCCTGATAAGGAACGATTGACATTAGAGTTGGAATCTCCAACAGTAACTGCAGTAACTCCAAAGGACCCAGTCTTAGCTGAGGTCACTGGGATGAAGGTCCTGCTTTCCTTCGTGTGTGGGTAATGAGAGGTATAACCTTCCAGTAAAGTAGTTGAGCCTTCAGAATGATTTGGGTCTCTTCTTCCACCTGTGACATCATTCCTATTGCTTGATGTCAGAGTAGAAGTTGTTGGATGGTCTTTATCTTCTTCCAAGCCTTCATGTGATGTAGAGAAGCTCTGAGAATTACTCTGCTGCGTTGTCATTGAAAGAGGTCCTGTCCTGTCCAAATCTTCCACCAAACCTGTGTTTGGATTTGCAGTANGCTGAAGCGTTATACTATGACTGGAGTCCATATCCATCCTTCTTCCTGCTTGATGACCTCGTCCCATGGGGTGTGAGATTGGGTTGAAGAAATCAGTCCAGGAACTGTCCTCTGGGCTTGGNNTTNTCCNTCCTTGCATTGGNTGGNTGGNATGAGCTGNNNTGCAGCTGTCCCTNTNNCGANGGNAGTCTTCTTNNGGNGNTTGGCGANNNCCNNCATGCNTCTGTGNNNNNNCTNNNNNNNGGNNNNCNGTTNTCNNGTACTACTNGNNNNNCNGATNNGNNTGNANAANNNNGGGNCNNNNNNNTNNCNNNNGNATGCNCANN

**>vHA-SP6_R_F05.ab1_rc (reverse complementary)**

NNTGNGCATNCNNNNGNNANNNNNNNGNCCCNNNNTTNTNCANNCNNATCNGNNNNNCNAGTAGTACNNGANAACNGNNNNCCNNNNNNNAGNNNNNNCACAGANGCATGNNGGNNNTCGCCAANCNCCNNAAGAAGACTNCCNTCGNNANAGGGACAGCTGCANNNCAGCTCATNCCANCCANCCAATGCAAGGANGGANAANNCCAAGCCCAGAGGACAGTTCCTGGACTGATTTCTTCAACCCAATCTCACACCCCATGGGACGAGGTCATCAAGCAGGAAGAAGGATGGATATGGACTCCAGTCATAGTATAACGCTTCAGCNTACTGCAAATCCAAACACAGGTTTGGTGGAAGATTTGGACAGGACAGGACCTCTTTCAATGACAACGCAGCAGAGTAATTCTCAGAGCTTCTCTACATCACATGAAGGCTTGGAAGAAGATAAAGACCATCCAACAACTTCTACTCTGACATCAAGCAATAGGAATGATGTCACAGGTGGAAGAAGAGACCCAAATCATTCTGAAGGCTCAACTACTTTACTGGAAGGTTATACCTCTCATTACCCACACACGAAGGAAAGCAGGACCTTCATCCCAGTGACCTCAGCTAAGACTGGGTCCTTTGGAGTTACTGCAGTTACTGTTGGAGATTCCAACTCTAATGTCAATCGTTCCTTATCAGGAGACCAAGACACATTCCACCCCAGTGGGGGGTCCCATACCACTCATGGATCTGAATCAGATGGACACTCACATGGGAGTCAAGAAGGTGGAGCAAACACAACCTCTGGTCCTATAAGGACACCCCAAATTCCAGAATGGCTGATCATCTTGGCATCCCTCTTGGCCTTGGCTTTGATTCTTGCAGTTTGCATTGCAGTCAACAGTCGAAGAAGGTGTGGGCAGAAGAAAAAGCTAGTGATCAACAGTGGCAATGGAGCTGTGGAGGACAGAAAGCCAAGTGGACTCAACGGAGAGGCCAGCAAGTCTCAGGAAATGGTGCATTTGGTGAACAAGGAGTCGTCAGAAACTCCAGACCAGTTTATGACAGCTGATGAGACAAGGAACCTGCAGAATGTGGACATGAAGATTGGGGTGTACCCATACGATGTTCCAGATTACGCTTAAGAATTCTGCAGATATCCATCACACTGGCGCCGCTCGANCANNCANNNNNNNNNNN

CD44v3-10_HA atggacaagttttggtggcacgcagcctggggactctgcctcgtgccgctgagcctggcg 60

vHA-SP6_R_F05.ab1_rc ------------------------------------------------------------ 0

CD44v3-10_HA cagatcgatttgaatataacctgccgctttgcaggtgtattccacgtggagaaaaatggt 120

vHA-SP6_R_F05.ab1_rc ------------------------------------------------------------ 0

CD44v3-10_HA cgctacagcatctctcggacggaggccgctgacctctgcaaggctttcaatagcaccttg 180

vHA-SP6_R_F05.ab1_rc ------------------------------------------------------------ 0

CD44v3-10_HA cccacaatggcccagatggagaaagctctgagcatcggatttgagacctgcaggtatggg 240

vHA-SP6_R_F05.ab1_rc ------------------------------------------------------------ 0

CD44v3-10_HA ttcatagaagggcacgtggtgattccccggatccaccccaactccatctgtgcagcaaac 300

vHA-SP6_R_F05.ab1_rc ------------------------------------------------------------ 0

CD44v3-10_HA aacacaggggtgtacatcctcacatccaacacctcccagtatgacacatattgcttcaat 360

vHA-SP6_R_F05.ab1_rc ------------------------------------------------------------ 0

CD44v3-10_HA gcttcagctccacctgaagaagattgtacatcagtcacagacctgcccaatgcctttgat 420

vHA-SP6_R_F05.ab1_rc ------------------------------------------------------------ 0

CD44v3-10_HA ggaccaattaccataactattgttaaccgtgatggcacccgctatgtccagaaaggagaa 480

vHA-SP6_R_F05.ab1_rc ------------------------------------------------------------ 0

CD44v3-10_HA tacagaacgaatcctgaagacatctaccccagcaaccctactgatgatgacgtgagcagc 540

vHA-SP6_R_F05.ab1_rc ------------------------------------------------------------ 0

CD44v3-10_HA ggctcctccagtgaaaggagcagcacttcaggaggttacatcttttacaccttttctact 600

vHA-SP6_R_F05.ab1_rc ------------------------------------------------------------ 0

CD44v3-10_HA gtacaccccatcccagacgaagacagtccctggatcaccgacagcacagacagaatccct 660

vHA-SP6_R_F05.ab1_rc ------------------------------------------------------------ 0

CD44v3-10_HA gctaccagtacgtcttcaaataccatctcagcaggctgggagccaaatgaagaaaatgaa 720

vHA-SP6_R_F05.ab1_rc ------------------------------------------------------------ 0

CD44v3-10_HA gatgaaagagacagacacctcagtttttctggatcaggcattgatgatgatgaagatttt 780

vHA-SP6_R_F05.ab1_rc ------------------------------------------------------------ 0

CD44v3-10_HA atctccagcaccatttcaaccacaccacgggcttttgaccacacaaaacagaaccaggac 840

vHA-SP6_R_F05.ab1_rc ------------------------------------------------------------ 0

CD44v3-10_HA tggacccagtggaacccaagccattcaaatccggaagtgctacttcagacaaccacaagg 900

vHA-SP6_R_F05.ab1_rc ------------------------------------------------------------ 0

CD44v3-10_HA atgactgcagatgtagacagaaatggcaccactgcttatgaaggaaactggaacccagaa 960

vHA-SP6_R_F05.ab1_rc ------------------------------------------------------------ 0

CD44v3-10_HA gcacaccctcccctcattcaccatgagcatcatgagga---agaagagaccccacattct 1017

vHA-SP6_R_F05.ab1_rc ---------------------NNTGNGCATNCNNNNGNNANNNNNNNGNCCCNNNNTTNT 39

** **** * * *** ** *

CD44v3-10_HA acaagcacaatccaggcaactcctagtagtacaacggaagaaacagctacccagaaggaa 1077

vHA-SP6_R_F05.ab1_rc NCANN-CNNATCNGNNN---NNCNAGTAGTACNNGANAACNG---NN-------NNCCNN 85

** *** * ******** **

CD44v3-10_HA cagtggtttggcaacagatggcatgagggatatcgccaaacacccaaagaagactcccat 1137

vHA-SP6_R_F05.ab1_rc NNNNNAGNNNNNNCACAGANGCATGNNGGNNNTCGCCAANCNCCNNAAGAAGACTNCCNT 145

***** ** ******* * ** ********* ** *

CD44v3-10_HA tcgacaacagggacagctgcagcctcagctcataccagccatccaatgcaaggaaggaca 1197

vHA-SP6_R_F05.ab1_rc CGNNA---NAGGGACAGCTGCANNNCAGCTCATNCCANCCANCCAATGCAAGGANGGANA 202

** ******** *** *** ************ *** *

CD44v3-10_HA acaccaagcccagaggacagttcctggactgatttcttcaacccaatctcacaccccatg 1257

vHA-SP6_R_F05.ab1_rc ANNCCAAGCCCAGAGGACAGTTCCTGGACTGATTTCTTCAACCCAATCTCACACCCCATG 262

* *********************************************************

CD44v3-10_HA ggacgaggtcatcaagcaggaagaaggatggatatggactccagtcatagtataacgctt 1317

vHA-SP6_R_F05.ab1_rc GGACGAGGTCATCAAGCAGGAAGAAGGATGGATATGGACTCCAGTCATAGTATAACGCTT 322

************************************************************

CD44v3-10_HA cagcctactgcaaatccaaacacaggtttggtggaagatttggacaggacaggacctctt 1377

vHA-SP6_R_F05.ab1_rc CAGCNTACTGCAAATCCAAACACAGGTTTGGTGGAAGATTTGGACAGGACAGGACCTCTT 382

**** *******************************************************

CD44v3-10_HA tcaatgacaacgcagcagagtaattctcagagcttctctacatcacatgaaggcttggaa 1437

vHA-SP6_R_F05.ab1_rc TCAATGACAACGCAGCAGAGTAATTCTCAGAGCTTCTCTACATCACATGAAGGCTTGGAA 442

************************************************************

CD44v3-10_HA gaagataaagaccatccaacaacttctactctgacatcaagcaataggaatgatgtcaca 1497

vHA-SP6_R_F05.ab1_rc GAAGATAAAGACCATCCAACAACTTCTACTCTGACATCAAGCAATAGGAATGATGTCACA 502

************************************************************

CD44v3-10_HA ggtggaagaagagacccaaatcattctgaaggctcaactactttactggaaggttatacc 1557

vHA-SP6_R_F05.ab1_rc GGTGGAAGAAGAGACCCAAATCATTCTGAAGGCTCAACTACTTTACTGGAAGGTTATACC 562

************************************************************

CD44v3-10_HA tctcattacccacacacgaaggaaagcaggaccttcatcccagtgacctcagctaagact 1617

vHA-SP6_R_F05.ab1_rc TCTCATTACCCACACACGAAGGAAAGCAGGACCTTCATCCCAGTGACCTCAGCTAAGACT 622

************************************************************

CD44v3-10_HA gggtcctttggagttactgcagttactgttggagattccaactctaatgtcaatcgttcc 1677

vHA-SP6_R_F05.ab1_rc GGGTCCTTTGGAGTTACTGCAGTTACTGTTGGAGATTCCAACTCTAATGTCAATCGTTCC 682

************************************************************

CD44v3-10_HA ttatcaggagaccaagacacattccaccccagtggggggtcccataccactcatggatct 1737

vHA-SP6_R_F05.ab1_rc TTATCAGGAGACCAAGACACATTCCACCCCAGTGGGGGGTCCCATACCACTCATGGATCT 742

************************************************************

CD44v3-10_HA gaatcagatggacactcacatgggagtcaagaaggtggagcaaacacaacctctggtcct 1797

vHA-SP6_R_F05.ab1_rc GAATCAGATGGACACTCACATGGGAGTCAAGAAGGTGGAGCAAACACAACCTCTGGTCCT 802

************************************************************

CD44v3-10_HA ataaggacaccccaaattccagaatggctgatcatcttggcatccctcttggccttggct 1857

vHA-SP6_R_F05.ab1_rc ATAAGGACACCCCAAATTCCAGAATGGCTGATCATCTTGGCATCCCTCTTGGCCTTGGCT 862

************************************************************

CD44v3-10_HA ttgattcttgcagtttgcattgcagtcaacagtcgaagaaggtgtgggcagaagaaaaag 1917

vHA-SP6_R_F05.ab1_rc TTGATTCTTGCAGTTTGCATTGCAGTCAACAGTCGAAGAAGGTGTGGGCAGAAGAAAAAG 922

************************************************************

CD44v3-10_HA ctagtgatcaacagtggcaatggagctgtggaggacagaaagccaagtggactcaacgga 1977

vHA-SP6_R_F05.ab1_rc CTAGTGATCAACAGTGGCAATGGAGCTGTGGAGGACAGAAAGCCAAGTGGACTCAACGGA 982

************************************************************

CD44v3-10_HA gaggccagcaagtctcaggaaatggtgcatttggtgaacaaggagtcgtcagaaactcca 2037

vHA-SP6_R_F05.ab1_rc GAGGCCAGCAAGTCTCAGGAAATGGTGCATTTGGTGAACAAGGAGTCGTCAGAAACTCCA 1042

************************************************************

CD44v3-10_HA gaccagtttatgacagctgatgagacaaggaacctgcagaatgtggacatgaagattggg 2097

vHA-SP6_R_F05.ab1_rc GACCAGTTTATGACAGCTGATGAGACAAGGAACCTGCAGAATGTGGACATGAAGATTGGG 1102

************************************************************

CD44v3-10_HA gtgTACCCATACGATGTTCCAGATTACGCTtaa--------------------------- 2130

vHA-SP6_R_F05.ab1_rc GTGTACCCATACGATGTTCCAGATTACGCTTAAGAATTCTGCAGATATCCATCACACTGG 1162

*********************************

CD44v3-10_HA ---------------------------- 2130

vHA-SP6_R_F05.ab1_rc CGCCGCTCGANCANNCANNNNNNNNNNN 1190
